# Relationship between sleep quality and marital satisfaction of working women during the premenopausal period

**DOI:** 10.1038/s41598-024-51440-w

**Published:** 2024-01-13

**Authors:** P. Yadollahi, S. Mavaddatnia, M. Zarshenas, P. Ghaemmaghami

**Affiliations:** 1 Department of Midwifery, Maternal-Fetal Medicine Research Center, School of Nursing and Midwifery, Shiraz University of Medical Sciences, Shiraz, Iran; 2 School of Nursing and Midwifery, Shiraz University of Medical Sciences, Shiraz, Iran; 3 Department of Midwifery, School of Nursing and Midwifery, Shiraz University of Medical Sciences, Shiraz, Iran

**Keywords:** Biophysics, Developmental biology

## Abstract

Sleep disorders can adversely affect physical, sexual, and marital health, particularly among middle-aged women. This study aimed to determine the relationship between sleep quality and marital satisfaction of working women during the premenopausal period. In this cross-sectional study, we selected 150 women working at Shiraz University of Medical Sciences in Iran was selected using random cluster sampling. A demographic information form, the *Pittsburgh Sleep Quality Index* (PSQI), and the *Evaluation and Nurturing Relationship Issues, Communication, and Happiness* (ENRICH) marital satisfaction scale were used for data collection. The Data were analyzed using SPSS.22 software at a significance level of *P* < 0.05. Multiple linear regression analysis was employed to predict sleep quality based on marital satisfaction. Our results showed that 79 (52.7%) of the participants had undesirable sleep quality, 87 (58%) had high marital satisfaction, and 32 (21.3%) had very high marital satisfaction. Regression analysis revealed that the total marital satisfaction score could not predict the sleep quality score of the participants. However, as dimensions of marital satisfaction, personality issues negatively (β = 0.327, *P* < 0.05) and ideological orientation positively (β = 0.336, *P* < 0.01) predicted the sleep quality score. Based on the prediction of the sleep quality score by personality issues and ideological orientations among the dimensions of marital satisfaction, it seems that life skills training, especially in these two dimensions, may improve the quality of sleep and, as a result, the physical and mental health of working women.

## Introduction

Sleep is a natural behavioral process involving reduced responses to external stimuli and changes in the activity of the cerebral cortex and muscle strength^1^. Every human spends about 27 years of his life sleeping, which alone expresses sleep’s importance^2^.

With aging comes reduced sleep quantity and quality, increasing the prevalence of insomnia^3^; hormonal changes, especially in sex steroids such as estrogen, progesterone, and testosterone, have substantial effects on brain functions such as cognition and the sleep–wake cycle^4^. During menopause, with reduced ovarian hormones and increased pituitary gonadotropins, women experience irregular menstrual and sleep–wake cycles; the sleep duration is short, and its quality becomes undesirable^1,5,6^. A significant number of premenopausal women refer to this period as a challenging period for sleep, such that the prevalence of sleep disorders during the climacteric period is 39–47%^7,8^.

Several factors affect the sleep quality of women. One of these factors is the sexual performance and relationship between couples^9^. Marital satisfaction strengthens couples’ relationships, gives them a sense of pleasure, and improves self-confidence, interpersonal relationships, and physical, sexual, and psychological health^10^. This is while some studies suggest that the quality of marital relations and marital satisfaction reduces with age^11^. Of course, this reduction is different between women and men; female sexual desire falls more steeply over time, which can cause a reduction in couples’ marital satisfaction^12^.

One study on sexual health in Iran have reported many problems in sexual relationships between Iranian couples, which may be one of the reasons for the increase in divorce rates in recent years^13^. Increasing of divorce especially emotional divorce is due to marital burnout is caused by a mismatch between the facts and expectations of the couple, and its severity depends on the compatibility of the couple and their beliefs. Physical marital burnout are characterized by symptoms such as fatigue, lethargy, chronic headaches, abdominal pain and sleep disturbances^14^. Meanwhile, the role of sleep disorders in the occurrence of sexual problems in couples during the premenopausal period is significant. Since middle-aged people also share their sleeping environment with their partners, sleep conditions and quality can affect their marital relationship**.** Some studies indicate that better sleep quality, longer sleep time, and less variability in sleeping and waking time positively impact people’s life satisfaction^15,16^. In recent years, due to the modernization of society, the change in the structure of women’s life and employment, and the increase in working hours of couples outside the home, the upbringing of children, working at home and greater fatigue during rest periods, sleep–wake cycle changes and sleep disorders have become prevalent. This issue significantly affect the physical, social, mental, and even sexual health. On the other hand, premenopausal women who experience the climacteric period suffer a series of physical and mental physiological changes and even sleep–wake cycle changes. Considering sleep problems faced by middle-aged women and their possible impacts on physical and sexual health as marital burnout and marital unsatisfaction and also few studies indicated this issue in this age group, we aimed to determine the relationship between sleep quality and marital satisfaction in premenopausal working women.

## Methods

This study is based on a cross-sectional study design and included all women working at Shiraz University of Medical Sciences, Shiraz, Iran, who were randomly selected through cluster sampling from January to April in 2021. In the absence of any similar prior research, while considering a 95% confidence level, an 80% test power, a moderat expected correlation coefficient of 0.3, assuming a design effect of 1.5, and a 20% dropout rate, the minimum required sample size has been estimated to be 150 individuals using G*Power (version 3.1) sample size calculation software.

The sample size was calculated based on the following formula:

$$C\left( r \right) = \frac{1}{2}Ln\frac{1 + r}{{1 - r}};\quad n = \frac{{\left( {z_{{\frac{\alpha }{2}}} + z_{\beta } } \right)^{2} }}{{\left[ {C\left( r \right)} \right]^{2} }} + 3$$

n represents the required sample size, $z_{{\frac{\alpha }{2}}}$ and $z_{\beta } { }$ denotes the Z-value for the significance level $\frac{\alpha }{2}$ and $\beta$ respectively. In addition , $r$ is the assumed sample correlation coefficient.

The sampling method employed in this study is a random cluster type. The study’s target population includes employed women working at Shiraz University of Medical Sciences in Iran who are in their premenopausal phase. Shiraz University of Medical Sciences is subdivided into seven clusters. A random selection process is utilized to pick a representative subset of clusters from the entire list. The number of clusters chosen is determined based on the desired sample size and the cluster size. From the sub-clusters, three specific faculties—Medicine, Nursing and Midwifery and Health and Pharmacy—were randomly selected. Within each selected cluster, an exhaustive list of employed women in their premenopausal phase was compiled. Random sampling was carried out within the chosen clusters, involving the selection of employed women in their premenopausal phase as participants for the study. This was achieved through simple random sampling. Data collection involved reaching out to and gathering data from the selected participants within each cluster through questinnare, with a specific focus on maternal satisfaction indicators and sleep quality. It was essential to maintain consistency and standardization in the data collection process across all clusters to ensure the study’s integrity. Following data collection, the information was subjected to analysis to draw conclusions and make inferences about the entire population. During this analysis, the clustering effect was taken into consideration to address potential biases.

The researchers visited these schools and selected those who met the study inclusion criteria by objective-based convenience. Eligible for inclusion were female employees of Shiraz University of Medical Sciences who were married, willing to participate, ≥ 40 years old, had not reached menopause, had not had a hysterectomy, did not have a severe marital problem, were not using drugs, alcohol, sleeping pills, or drugs that affect sleep quality and quantity (antidepressants, some appetite suppressants such as Liraglutide and some cardiac drugs such as propranolol, amiodarone, carvedilol, etc.), had no history of mental diseases and were not using psychiatric drugs, and had not experienced an uncomfortable or stressful event in the past six months, such as the death of a family member. The study exclusion criteria were not answering more than 20% of the items and wishing to withdraw from the study at any time.After receiving permission from the Institutional Ethics Committee (IR.SUMS.REC.1399.490), the researchers explained the study’s objectives to potential subjects and informed them that participation in this study was optional, the questionnaires were anonymous, and all the information recorded was confidential. Finally, those who wished to participate filled out an informed consent form before receiving the questionnaire. Then, the participants filled out the questionnaire during their work shifts.

A demographic information form, four-point Likert scale of “*Pittsburgh Sleep Quality Index”* (PSQI), and the “*Evaluation and Nurturing Relationship Issues, Communication, and Happiness”* (ENRICH) marital satisfaction scale were used for data collection. The PSQI was designed and psychometrically studied by Buysse et al. (1989) at the Pittsburgh Institute of Psychiatry with nine questions across seven dimensions: the subjective quality of sleep, delay in falling asleep, duration of useful sleep, adequacy of sleep, sleep disorders, the use of sleep-inducing drugs, and disruption in daily functioning. These items are scored on a four-point Likert scale between 0,1,2 and 3 indicated the normal, mild, moderate and severe condition respectively.The range of the total score is 0–21. Scores above six indicate undesirable sleep quality. Validity and realebility of PSQI in Iran estimated as Chronbach’s alpha coefficient was 0.55. KMO value was 0.58, and it was significant at 0.05^16,17^.

Olson, Furnier, and Druckman designed the ENRICH Marital Satisfaction Scale. Its original version has 125 questions and 12 subscales^18^. Suleimanian et al. have prepared its shortened form with 47 questions across nine dimensions^19^, scored on a five-point Likert scale from 1 (completely disagree) to 5 (completely agree). The ENRICH marital satisfaction scale, designed by David H. Olson, assesses marital satisfaction across nine dimensions: Personality issues, Marital relationship, Marital conflict, sexuality, financial management, leisure activities, children and parenting, Ideological orientation and family and friends^18^.

In the scale questions such as 1, 2, 3, 5, 7, 9, 10, 17, 25–29, 34–36 and 43 are scored based on Likert scale. But questions 4, 6, 8, 11–16, 18–24, 30–33, 37–42, and 45–47 are scored in reverse. The total score ranges between 47 and 235. Scores between 47 and 84 indicate high dissatisfaction, scores between 85 and 122 indicate relative dissatisfaction, 123–160 indicate moderate satisfaction, 161–198 indicate high satisfaction and 199–235 indicate very high satisfaction^19,20^.

Data were analyzed using descriptive (mean, standard deviation, and quantitative and qualitative description of variables) and inferential (multiple linear regression) statistics by SPSS version 22. In the evaluation of the appropriateness of a linear regression model, a range of diagnostic measures was employed. The R-squared value, a widely used metric for assessing goodness of fit, was elucidated to convey the proportion of variance explained by the independent variables. Furthermore, attention was directed towards the adjusted R-squared, which takes into account the number of predictors in the model. Subsequently, the analysis will extend to the F-statistic, a tool that examines the overall significance of the regression model. Multicollinearity was also addressed through the use of the Variance Inflation Factor (VIF), with calculated values consistently below the threshold of 10***.*** For all statistical analyses, a significance level of < 0.05 was considered.

### Declaration of Helsinki

All methods were performed in accordance with the relevant guidelines and regulations.

## Results

In Table 1, demographic features of the sample are listed, and their correlation with sleep quality has been examined to identify the confounding variable. This study included 150 female participants with an average age of 51.44 ± 1.3 years. The average years of marriage were 17.21 ± 5.51. Most participants had a bachelor’s degree (67.8%), were employees (100%), had an employed husband (85.2%), and had two children (68.3%). In addition, most of their spouse’s education levels and monthly incomes were Ph.D. or above (44.3%) and more than 100 million IRR (48.6%), respectively.

**Table 1:** Sample characteristics and test with sleep quality.

Characteristics	Levels	N (%)	Mean ± SD	Test	Test value	*P* value
Qualitative variables						
Education	Bachelor’s degree	32 (21.3)	6.41 ± 4.38	ANOVA	F = 0.144	0.866
	Master’s degree	101 (67.3)	6.03 ± 4.01			
	Ph.D	17 (11.3)	5.82 ± 3.81			
Partner education	Undergraduate level and below	36 (24)	7.33 ± 5.06	ANOVA	F = 2.653	0.074
	Master’s degree	48 (32)	6.06 ± 3.48			
	Ph.D	66 (44)	5.42 ± 3.69			
Job	Employee	97 (64.7)	5.76 ± 3.99	T-Test	T = -1.33	0.186
	Non-academic staff	53 (35.3)	6.68 ± 4.12			
Partner job	Employee	59 (39.3)	6.356 ± 4.46	ANOVA	F = 1.571	0.199
	Non-academic staff	68 (45.3)	5.60 ± 3.28			
	Academic Staff	9 (6)	5.11 ± 4.11			
	Non-dual employment	14 (9.3)	7.93 ± 5.21			
Income	Less than 5 million	11 (7.3)	4.64 ± 4.27	ANOVA	F = 2.817	0.063
	Between 5 and 10 million	67 (44.7)	6.91 ± 4.24			
	More than 10 million	72 (48.0)	5.54 ± 3.71			
Child number	1	35 (23.3)	6.80 ± 3.98	ANOVA	F = 2.563	0.08
	2	98 (65.3)	5.57 ± 3.82			
	3	17 (11.3)	7.59 ± 5.00			
Quantitative variables						
Age	147	44.40	3.09	Spearman	r = 0.080	0.335
Marriage duration	149	17.23	5.49	Spearman	r = 0.059	0.475

N, number of cases in total; SD, standard deviation; ANOVA, One-way analysis of variance; T-Test, Independent Samples t-Test; r, Correlation coefficient.

The results of the quantitative evaluation of sleep quality score and marital satisfaction are shown in Table 2.

**Table 2:** Quantitative evaluation of sleep quality and marital satisfaction.

Variables	Number of Item	Score Range	Mean	Median	SD
Independent variables					
Personality issues	5	5–25	19.03	19	3.76
Marital relationship	6	6–30	19.44	20	3.31
Marital conflict	6	6–30	22.19	22	3.68
Financial management	5	5–25	19.99	20	3.28
Leisure activities	5	5–25	17.55	18	3.34
Sexuality	6	6–30	26.51	27	4.215
Children and parenting	5	5–25	19.69	20	3.46
Family and friends	5	5–25	19.33	20	3.135
Ideological orientation	4	4–20	20.45	20	3.29
Total	47	47–235	179.81	181	24.19
Dependent variables					
Sleep quality	9	0–21	6.09	6	4.05

SD, standard deviation.

The qualitative description of sleep disorder scores and marital satisfaction is presented in Table 3. In the qualitative assessment of sleep disorder scores, it was observed that sleep disorders were distributed approximately equally between desirable and undesirable sleep. Additionally, the majority of women, 87 (58%), reported high marital satisfaction, while 32 (21.3%) reported very high marital satisfaction.

**Table 3:** Qualitative description of sleep disorder score and marital satisfaction.

Variables	Range	N	%
Sleep disorder score			
Undesirable	6–21	79	52.7
Desirable	0–5	71	47.3
Marital satisfaction			
High dissatisfaction	47–84	1	0.7
Relative dissatisfaction	85–122	1	0.7
Moderate satisfaction	123–160	29	19.3
High satisfaction	161–198	87	58
Very high satisfaction	199–235	32	21.3

N, number of cases in total; %, Percent of number.

Multiple linear regression analysis was used to predict sleep quality by marital satisfaction in Table 4. The R-squared value revealed that approximately 53% of the variance in the dependent variable was accounted for by the independent variables. Moreover, the adjusted R-squared offered a detailed evaluation of the model’s explanatory capability. The significance of the F-statistic (F(9,138) = 2.3, *P* = 0.019) affirmed the overall significance of the regression. Assessment of multicollinearity using the Variance Inflation Factor (VIF) indicates the absence of problematic correlations among predictors. In conclusion, the linear regression model demonstrated a satisfactory fit. As shown in Table 4, among sub-dimensions of marital satisfaction, the two dimensions of personality issues (β = 0.327, *P* = 0.05) negatively and ideological orientation (β = 0.336, *P* = 0.013) positively predicted poor sleep quality scores. That is, the higher the understanding of personality between couples, the better the wife’s sleep quality, and the greater the conflict in ideological orientations, the worse the wife’s sleep quality.

**Table 4:** Result of the multiple regression model with sleep quality as the outcome variable.

Predictors	B	SE	β	T value	*P* value	95.0% CI for B		VIF
						Lower	Upper	
(Intercept)	8.71	2.569		3.391	.001	3.633	13.793	3.796
Personality issues	− 0.327	0.168	− 0.302	− 1.950	0.05	− 0.659	0.005	5.371
Marital relationship	0.004	0.227	0.003	0.018	0.986	− 0.445	0.453	3.894
Marital conflict	− 0.122	0.174	− 0.110	− 0.702	0.484	− 0.466	0.221	2.283
Financial management	0.037	0.149	0.029	0.246	0.806	− 0.257	0.330	2.176
Leisure activities	− 0.073	0.142	− 0.060	− 0.512	0.610	− 0.353	0.208	2.930
Sexuality	− 0.112	0.130	− 0.116	− 0.857	0.393	− 0.370	0.146	2.428
Children and parenting	0.189	0.145	0.161	1.301	0.195	− 0.098	0.476	2.361
Family and friends	− 0.045	0.158	− 0.034	− 0.282	0.778	− 0.357	0.268	1.860
Ideological orientation	0.336	0.134	0.272	2.512	0.013	0.072	0.600	3.796

B, unstandardized coefficients regressin estimate; SE, standard error; β, standardized coefficients regressin estimate; CI, confidence interval; VIF, variance inflation factor.

## Discussion

The present study revealed that the total marital satisfaction score does not predict the sleep quality score of premenopausal working women. In explaining this relationship, one can say daily occupational status and not safe physical activity of these women make it difficult to achieve high sleep quality. However, among the dimensions of marital satisfaction, personality issues negatively and conflict in the ideological orientation of couples positively predicted poor sleep quality, i.e., the more the understanding of personality between couples, the lower the poor sleep quality score. Consistent with the present results, Sassoon et al. showed a positive relationship between the neurotic personality of women during the premenopausal period and sleep disorders^21^. Brigitte et al. showed that compatible and agreeable people had better sleep time and quality^22^. Stephan et al. stated that extroverted people have better sleep quality^23^.

Our results indicated that conflicts in ideological orientations between couples predict decreased sleep quality. It seems these conflict lead to continuse tention between couple and so lack of peace and high sleep quality. In this regard, Hill et al. stated that adults with religious beliefs had healthier and better sleep quality outcomes than their less religious counterparts, and doubts about ideological orientations and less belief in religious issues had an inverse relationship with sleep quality^24^.

The present study found that most participants had undesirable sleep quality. Consistent with our findings, Cibelle et al. reported that women had a worse sleep quality during the climacteric period than during menstruation and experienced mild to moderate insomnia^25^. Jones et al., in their study on sleep problems of middle-aged women during the premenopausal period, showed that most women had relatively poor sleep quality^26^. Lampio et al. linked the premenopausal period with reduced total sleep time and efficiency, waking up after sleeping, and waking up every hour^27^. In contrast, Wenjun et al. found that the sleep quality of premenopausal women was better than post-menopausal women and induced menopause^28^. Jahangiri et al. also stated that most non-menopausal and menopausal women did not report any sleep disorders^29^. Such discrepancies can be attributed to differences in culture, economic status, number of children, years of marriage, underlying diseases, exercise and nutrition, marital problems, and other factors.

Our results indicated high marital satisfaction in most participants. Shareh et al. also found that the marital satisfaction of middle-aged women was high^30^, while Talaizadeh et al. reported that marital satisfaction was almost the same in different age groups^31^. Thus, based on the present results, it seems that factors other than age are effective on the marital satisfaction of women during this period, the investigation of which was not one of the objectives of this study. Finally, it can be said because of biological and individual differences these findings cannot be generalized to all adultery in the premenstrual period.

### Limitations and strengths

One of the limitations of the study is not examining some factors that probably affect sleep quality such as body mass index, physical activity, and nutrition in these people, and examining the relationship of these variables with sleep quality and, if necessary, controlling their confounding effect. The present study’s strength was dealing with the sleep quality of working women and its relationship with marital satisfaction, which can help plan solutions to improve women’s sexual and mental health.

## Conclusion

The study results showed that more than half of the working women during the climacteric period had undesirable sleep quality. At the same time, they reported high marital satisfaction scores. Although the marital satisfaction score could not predict the sleep quality of working women, some of its dimensions, namely personality issues and ideological orientations of couples, could predict the sleep quality. Therefore, it seems that life skills training, especially in these two dimensions, may improve the quality of sleep and, as a result, the physical and mental health of working women during the premenopausal period.

The protocol of the current study was approved by the ethics committee of the Shiraz University of Medical Sciences (No: IR.SUMS.REC.1399.490) and informed consent was received from each participant.

## Data Availability

All respectable readers and researchers can request the data by directly contacting the primary author at Ghaemmaghami nursing.midwifery.school@gmail.com.

## Abbreviations

PSQI: Pittsburgh Sleep Quality Index
ENRICH: Evaluation and Nurturing Relationship Issues, Communication, and Happiness
